# Supplementary material for: Amphiphilic Polyethylene-b-poly(L-lysine) Block Copolymer: Synthesis, Self-Assembly, and Responsivity
Source: Int J Mol Sci. 2023 Mar 13;24(6):5495. doi: 10.3390/ijms24065495 (PMC10052655; doi:10.3390/ijms24065495)
Supplement: Supplementary file 1 [file ijms-24-05495-s001.zip › ijms-2258847-supplementary.pdf]

International Journal of *Molecular Sciences*

MDPI

Supplementary Materials

Amphiphilic Polyethylene-*b*-poly(L-lysine) Block Copolymer: Synthesis, Self-assembly, and Responsivity

Lixia Pei [1], Hongyu Ma [2], Yan Jiang [2], Handou Zheng [3] and Haiyang Gao [3,*]

[1] School of Chemistry and Chemical Engineering, South China University of Technology, Guangzhou 510641, China
[2] Daqing Chemical Engineering Research Center, Petrochemical Research Institute, Daqing 163714, China
[3] School of Materials Science and Engineering, PCFM Lab, GD HPPC Lab, Sun Yat-sen University, Guangzhou 510275, China
* Correspondence: gaohy@mail.sysu.edu.cn

General Procedures

All manipulations involving air- and moisture- sensitive compounds were carried out under an atmosphere of dried and purified nitrogen with standard vacuum-line, Schlenk, or glovebox techniques.

Materials

Dichloromethane and chloroform were distilled from CaH_2 under nitrogen, and toluene and hexane from Na/K alloy. Ethylene (99.99%) was purified by passing through Agilent moisture and oxygen traps. Amine-imine nickel complex [Ar-N=C(Me)-$(Me)_2$C-NH-Ar]$NiBr_2$ (Ar = 2,6-diisopropylphenyl) was synthesized according to our previously reported method. MMAO (7 wt. % Al in heptane) was purchased from Akzo-Nobel and used as received. $ZnEt_2$ was purchased from Aldrich and used as received. ε-Benzyloxycarbonyl-L-lysine-*N*-carboxyanhydride (Z-Lys-NCA) was purchased from Acros and was recrystalized from hexane/ethyl acetate mixture solvent three times to afford pure polymerization-grade NCA monomer. All solvents and other reagents if not specified were directly purchased and used as received.

Synthesis of PE–OH

In a typical procedure, a round-bottom Schlenk flask with stirring bar was heated 3 h to 150 °C under vacuum and then cooled to room temperature. The flask was pressurized to 1 atm of ethylene and vented three times. The 200 equiv. MMAO solution as a cocatalyst was introduced into the glass reactor under 0.2 atm of ethylene. The system was maintained by continuously stirring for 5 min, and then toluene and 2 mL solution of amine-imine nickel complex (100 μmol) in CH_2Cl_2 were syringed into the well-stirred solution in order, and the total reaction volume was kept at 60 mL. The ethylene pressure was kept a constant value of 0.2 atm throughout the reaction. The reaction temperature of 20 °C was maintained in polymerization experiment. After 15 minutes, the ethylene feed was stopped, and the reaction flask was charged with N_2 and vented for three times. Then $ZnEt_2$ solution (Zn/Ni=300) was charged into reaction system, and the system was maintained by continuously stirring for 3 h at room temperature. Dry O_2 was introduced into solution, and oxidative workup was performed at 100 °C for 2 h. Finally, the reaction solution was poured into acidic methanol (95:5 methanol/HCl). The resulting precipitated polymers were collected and treated by filtration, washing with methanol several times, and drying in vacuum at 40 °C to a constant weight. ^{1}H NMR ($CDCl_3$, ppm): 3.65 (t, 2H); 1.4-0.5 (PE).

Synthesis of PE–COOCH(^{i}Pr)NH_2

A dried flask containing 0.5 g PE–OH and 0.05 g *N*-*tert*-butoxycarbonyl-L-valine (BOC-L-valine) dissolved in 40 mL toluene was treated with a solution of 0.05 g DCC for 72 h at room temperature. Then, the dicyclohexylurea was removed by filtration. The polymer solution in the toluene was precipitated when poured into an excess of cold ethanol. The resulting precipitated polymer (PE–COOCH(^{i}Pr)NH–BOC) (0.46 g) was collected and purified by filtration, washing with ethanol several times, and drying in vacuum at 40 °C. ^{1}H NMR ($CDCl_3$, ppm): 4.15 (t, 2H); 1.46 (s, 9H); 1.4-0.5 (PE and CH(C*H*$_3$)$_2$).

The obtained PE–COOCH(^{i}Pr)NH–BOC was dissolved in 30 mL toluene. The solution was treated with 15 mL trifluoroacetic acid (TFA) for 2 h. TFA was then removed in vacuum, the residue was dissolved in 30 mL toluene and saturated aqueous $NaHCO_3$ and water. The polymer was precipitated when its toluene solution was poured into an excess of ethanol. The resulting precipitated polymer (PE–COOCH(^{i}Pr)NH_2) (0.41 g) was collected and purified by filtration, washing with ethanol several times, and drying in vacuum at 40 °C. ^{1}H NMR ($CDCl_3$, ppm) 4.14 (t, 2H); 1.4-0.5 (PE and CH(C*H*$_3$)$_2$).

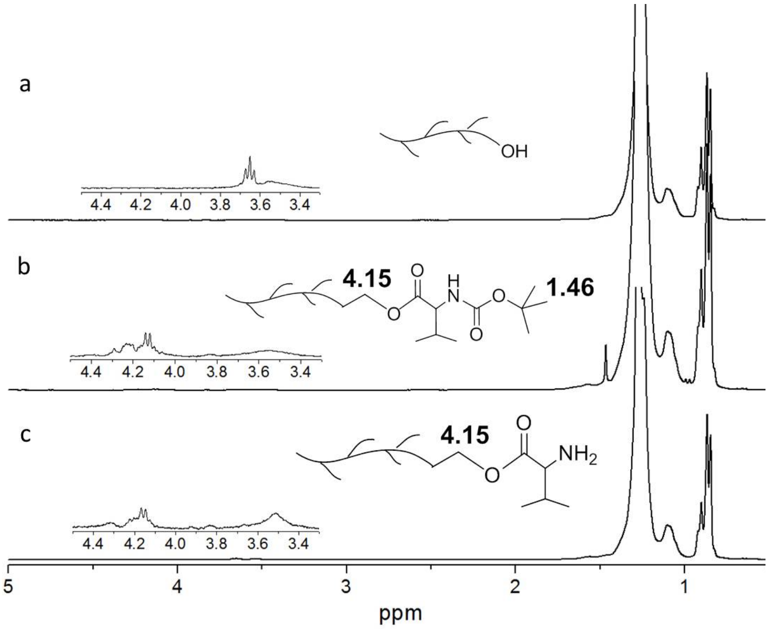

Figure S1. ^{1}H NMR spectra of (a) PE_{607}–OH, (b) PE_{607}–Val-Boc, and (c) PE_{607}–NH_2.

Table S1. DLS characterizations of PE_{607}-*b*-PLL_{275} polymeric micelles self-assembled in water at different pH values (Figure 6B).

Entry	pH	Average R_h (nm)	PDI	Peak value of R_h (nm)
1	1.5	97±1	0.140	102
2	6.2	117±2	0.112	115
